# Constructing protective mechanism by analysis of vaccine impact based on hybrid model of dynamic DEA and cluster tendency

**DOI:** 10.1186/s12889-025-24277-9

**Published:** 2025-10-08

**Authors:** Tzu-Jen Hung, Chao-Yang Kuo, Hung-Da Dai, Yi-Tui Chen, Emily Chia-Yu Su

**Affiliations:** 1 Shin Kong Wu Ho-Su Memorial Hospital, Taipei, 111 Taiwan; 2 Smart Healthcare Interdisciplinary College, National Taipei University of Nursing and Health Sciences, Taipei, 112 Taiwan; 3 Department of Nursing, Taipei Veterans General Hospital, Taipei, 112 Taiwan; 4 Department of Education and Research, Taipei City Hospital, Taipei, 103 Taiwan; 5 Department of Health Care Management, National Taipei University of Nursing and Health Sciences, Taipei, Taiwan; 6 Institute of Biomedical Informatics, College of Medicine, National Yang Ming Chiao Tung University, Taipei, 112 Taiwan; 7 Graduate Institute of Biomedical Informatics, College of Medical Science and Technology, Taipei Medical University, New Taipei City, 235 Taiwan; 8 Clinical Big Data Research Center, Taipei Medical University Hospital, Taipei, 110 Taiwan

**Keywords:** Undesirable output model, Dynamic data envelopment analysis, Epidemic prevention, Vaccination rate, Mortality

## Abstract

**Background:**

COVID-19 was announced as a public health emergency of international concerns by WHO in January 2020 and many global scientists started to develop vaccines to stop the spread of COVID-19. This study aims to analyze the effectiveness of vaccination to confirmed cases and deaths.

**Methods:**

This paper analyzed the impact of vaccines on COVID-19 from 17 selected countries and considered the impact of vaccines in epidemic prevention for evaluating the efficiency of epidemic prevention by establishing three scenarios. Firstly, we used the undesirable output model to analyze the impact of the number of confirmed cases and death as undesirable output to assess the policy effect. Then, we considered the vaccination rate as a carryover and used dynamic Data Envelopment Analysis to evaluate efficiency. Finally, the mortality variable was excluded to examine the impact of vaccines on mortality.

**Results:**

17 countries were classified via clustering analysis into three group based on elbow point plot. There are two countries ranked the best performance based on dynamic Data Envelopment Analysis in each group. It indicated that the countries with the same characteristics can improve their performance in preventing pandemic by learning the countries with similar characteristics.

**Conclusion:**

The vaccine administration is the main strategy for epidemic prevention. Furthermore, preventive measures are essential. In the post-epidemic era, government should reduce the mortality rate and main the medical system to ensure public welfare.

**Supplementary information:**

The online version contains supplementary material available at 10.1186/s12889-025-24277-9.

## Introduction

COVID-19 was announced as a public health emergency of international concerns by WHO in January 2020. High transmission and fatality rate are two main reasons to cause a great concern. On January 30, 2020, the World Health Organization (WHO) declared COVID-19 the sixth public health emergency of international concern [1]. To date, the COVID-19 pandemic continues to pose a significant threat to global health, and measures taken to curb its spread have resulted in a widespread slowdown of economic and industrial activities worldwide. The international industrial supply chain has also experienced severe disruptions beyond what was anticipated; consequently, global economic growth has been severely impacted, leading to sharp recessions in various economies worldwide. Moreover, the prolonged pandemic has caused a sense of epidemic fatigue and led to periodic spikes in the number of cases. It is often impossible to provide timely drug treatments or preventive vaccines for a sudden infectious disease, and the performance of COVID-19 epidemic prevention was not greatly improved until vaccines were developed [2].

Many global scientists started to develop vaccines to stop the spread of COVID-19. In December 2020, the vaccines Moderna, Pfizer BioNTech, and Johnson & Johnson were granted emergency use authorization (EUA) by Food and Drug Administration (FDA); furthermore, the AstraZeneca developed by Oxford was approved for use in UK as well. In May 2021, the global community reached a milestone in one billion COVID-19 vaccinations. The monthly new deaths went down significantly since then (shown in. 1).

**Fig. 1:**
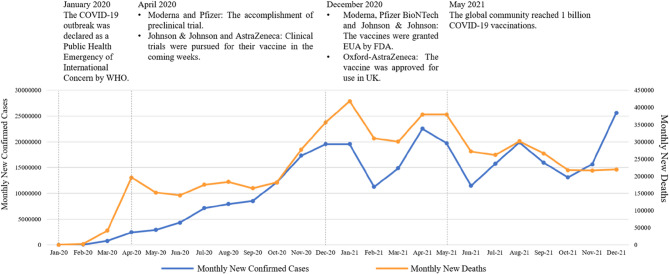
Time series of COVID-19 monthly new confirmed cases and monthly new deaths

After vaccines were developed, the government expected that increased vaccination rates may slow the epidemic; however, “vaccine hesitancy” hindered the promotion of vaccines and delayed the process of herd immunity [3, 4]. When there were huge disparities in the global vaccination rates, the public’s skeptical attitude towards vaccines, whether minors should be vaccinated, whether vaccines can prevent virus transmission, and other related issues slowed down the vaccination speed and hindered global herd immunity, but the efficacy of vaccines in blocking virus transmission was still recognized [2, 5, 6].

Some researches propose that vaccines can decrease the burden of healthcare system, improve the healthcare capacity, and help reducing the overall fatality rate [7–9]. However, the Watson et al. also pointed out that lower-middle-income and low-income countries have lower estimated deaths averted by vaccines compare to high-income and upper-middle-income countries. Moreover, low-income counties have lower impacts due to the lower coverage of vaccine [10].

In recent years, the spread of many outbreaks has caused major public health events and instability in many countries, seriously endangering people’s health and even significantly affecting economic development [11, 12]. Similar major public health events will cause global panic in the future, thus, how to prevent and control an epidemic as quickly as possible and prevent its spread as soon as possible are important issues. With advances in medical treatment and technology, such as vaccines, great progress has been made in epidemic prevention and control. However, epidemic prevention measures will have different effects due to the differences between the vaccine application rates and epidemic prevention policies of various countries. Therefore, it has become an important issue to determine the benchmark units with good epidemic prevention performance and establish epidemic prevention measures to contain an epidemic as soon as possible.

Data Envelopment Analysis (DEA) has been used in numerous articles to evaluate the effectiveness of epidemic prevention. Mohanta et al. measured the number of con-firmed cases and the number of recovered cases as performance indicators to explore the utilization of medical resources during COVID-19, as a performance indicator for management, and as a guideline for policy formulation. Yousefi et al. explored how to improve the effectiveness of healthcare services and efficiently allocate healthcare resources to a large number of confirmed cases within the relatively limited number of healthcare resources during COVID-19, and used Artificial Neural Network (ANN) to measure the relationship between future medical resource utilization and output to predict the overall efficiency and the ranking of treatment centers to provide a reference for future medical decision-making [13, 14]. In order to create a tool for management policies [15], Pourmahmoud and Bagheri divided efficiency into efficiency change and technology change, and then, used the Malmquist Index to discuss the changes of efficiency over time and evaluate the efficiency improve-ments of the medical care system during the epidemic period. Therefore, Data Envelopment Analysis was used to analyze the effectiveness of controlling COVID-19 epidemic. Then, the vaccination rate was taken into account as a carryover in the model to evaluate the efficiency of epidemic prevention of each country after considering the vaccination rate. Finally, we also exclude the number of confirmed cases and assess the impact of vaccines on the mortality rate. Cluster analysis was used to divide these countries into different group with similar characteristics, so that during efficiency improving process, better learning outcomes can be achieved due to the same characteristics among countries.

## Materials and methods

The number of confirmed cases, deaths, doctors, and nurses was derived from public reports from the World Health Organization. For the purpose of ensuring the integrity of the study data regarding the number of confirmed cases and deaths, the monthly data collected from December 2020 to December 2021 are included in this study. A total of 17 countries were included in this study, including 16 G20 countries. G20 is a forum held annually among the leaders of the world’s largest and fastest-growing countries. The G20 countries account for 85% of global GDP, thus, the G20 has become a new core of global economic power, which not only dominates the global political and economic situation, but also affects the resource allocation of countries around the world. In addition to the G20 countries, the three countries that were most seriously affected during the epidemic were also included. Spain experienced an alarming increase in the number of confirmed cases and deaths, and were listed among the countries with a “Level 3 Warning” for global travel, meaning travelers were advised to avoid nonessential travel to the countries.

This paper uses DEA to construct three models to analyze the efficiency of epidemic prevention. Firstly, we use the undesirable output model to analyze the impact of confirmed cases and treat death as undesirable output to assess the policy effect. Then, we consider the vaccination rate as a carryover and use dynamic Data Envelopment Analysis to evaluate efficiency. Finally, the number of confirmed cases is excluded to examine the impact of vaccines on mortality. We treat the number of doctors and of nurses as input, and the epidemic recovery is output. The number of confirmed cases, deaths are treated as undesirable output and the vaccination rates is carryover that may affect the confirmed case in the next period. Chen et al. [16] propose the epidemic severity to evaluate the transmission of COVID-19 and we transfer the epidemic severity into epidemic recovery to be an output for DEA model by Eq. (1). The monthly data collected from December 2020 to December 2021 are included in this study and a total of 17 countries were included in this study. Epidemic recovery $R_i$ is measured by the proportion of confirmed cases N to population P each day, expressed as

1 $$\:R_{\mathit i}\mathit=\mathit1\mathit-\frac{{\mathit N}_{\mathit i}}{\mathit P{\mathit t}_{\mathit i}}$$

**Fig. 2:**
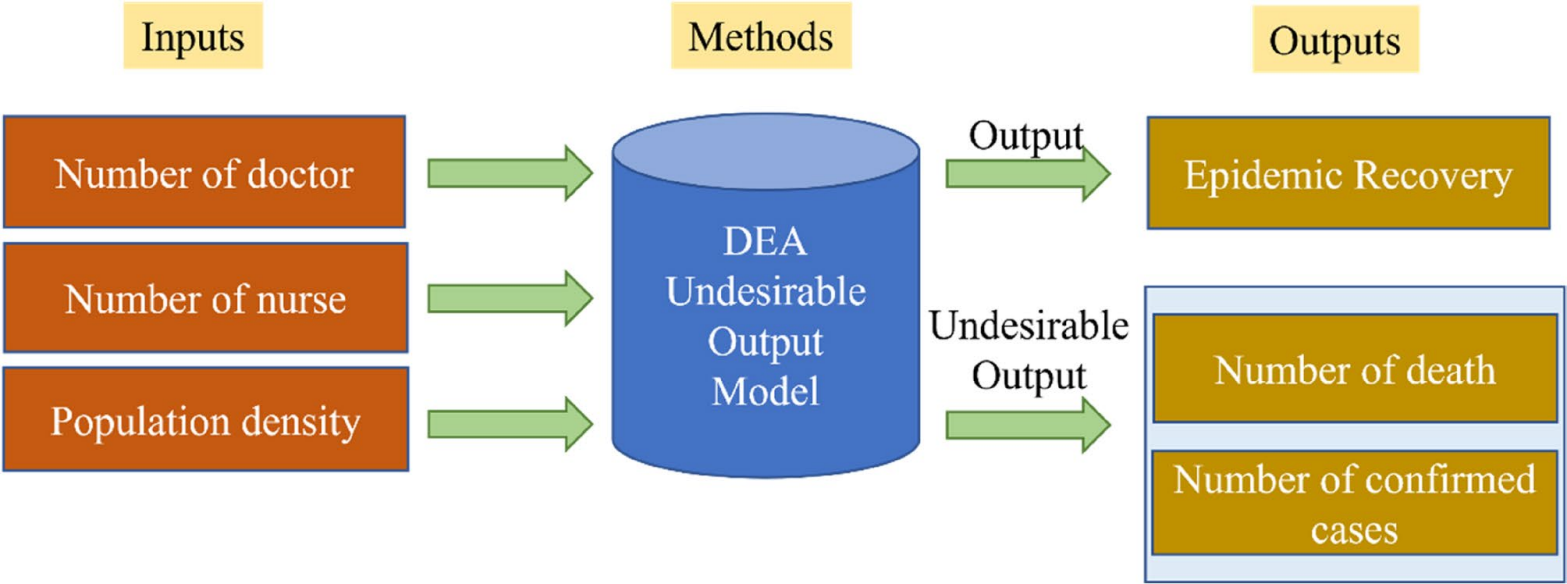
The DEA model with undesirable outputs of Model 1

The DEA model was used to evaluate the efficiency of epidemic prevention and this paper also considers the effect of vaccination to control the outbreak of COVID-19. Charnes et al. [17] proposed DEA based on concept of the frontier production function defined by Farrell [18] is a nonparametric method for measuring the relative efficiency between decision-making units (DMUs). The mitigation of the COVID-19 transmission in each country was executed by a technology whereby N countries in terms of DMUs transform multiple inputs $\:x=$ ($\:{x}_{1}$, …, $\:{x}_{m}$) $\:\in\:{\mathbb{R}}_{+}^{m}$ into multiple outputs $\:y=$ ($\:{y}_{1}$, …, $\:{y}_{s}$) $\:\in\:{\mathbb{R}}_{+}^{s}$. In Model 1 (shown in Fig. 2), we treat the number of doctors, the number of nurses and population density as input, and the epidemic recovery as output. However, considering the number of confirmed cases and the number of death areas inevitable and undesirable output, we adopt Undesirable DEA proposed by Tone to measure the efficiency of epidemic prevention [19]. The formula is following:

2 $$\:\text{min}\:\rho\:=\frac{1-\frac1{m{\sum\:}_{i=1}^m\frac{s_i^-}{x_{i0}}}}{1+\frac1{s_1+s_2}({\sum\:}_{r=1}^{s_1}\frac{s_r^g}{y_{r0}^g}+{\sum\:}_{r=1}^{s_2}\frac{s_r^b}{y_{r0}^b})}$$

$$\:\text{s}.\text{t}.\:\:{x}_{i0}={\sum\:}_{j=1}^{n}{\lambda\:}_{j}{x}_{ij}+{s}_{i}^{-},\:\:i=1,\dots\:,m$$

$$\:{y}_{{r}_{{1}^{0}}}^{g}={\sum\:}_{j=1}^{n}{\lambda\:}_{j}{y}_{{r}_{1}j}^{g}-{s}_{{r}_{1}}^{g},\:\:{r}_{1}=1,\dots\:,{s}_{1}$$

$$\:{y}_{{r}_{{2}^{0}}}^{b}={\sum\:}_{j=1}^{n}{\lambda\:}_{j}{y}_{{r}_{2}j}^{b}+{s}_{{r}_{2}}^{b},\:\:{r}_{2}=1,\dots\:,{s}_{2}$$

$$\:{s}_{i}^{-}\ge\:0,\:{s}_{{r}_{1}}^{g}\ge\:0,\:{s}_{{r}_{2}}^{b}\ge\:0,\:\:{\lambda\:}_{j}\ge\:0,\:j=1,\dots\:,n$$

where *x* is input, $\:{y}^{g}$ is good output and $\:{y}^{b}$ is undesirable output, $\:{\lambda\:}_{j}$ is a positive vector, the vectors $\:{s}_{i}^{-}$ is the input excess of the above formula, and $\:{s}^{g}$ and $\:{s}^{b}$ are the output shortfall of $\:{y}^{g}$ and $\:{y}^{b}$ of the above formula, respectively.

**Fig. 3:**
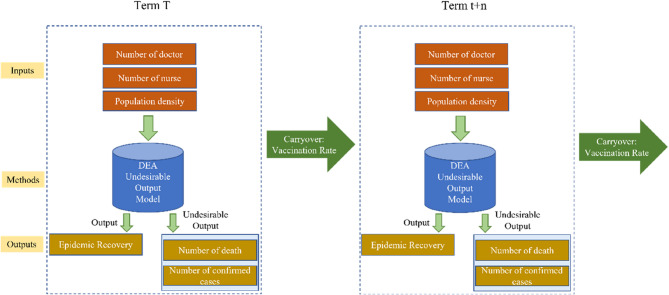
The structure of dynamic DEA model for Model 2

**Fig. 4:**
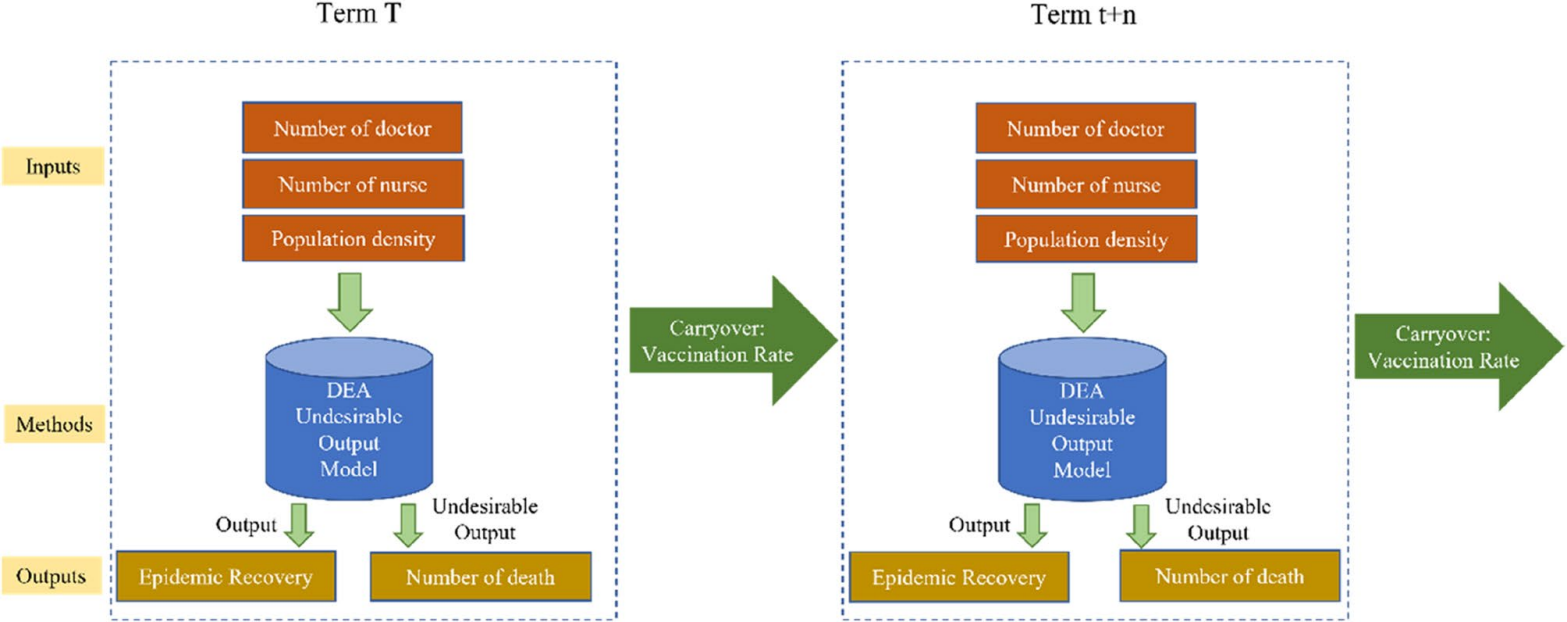
The structure of dynamic DEA model for Model 3

In order to analyze the effect of vaccine to efficiency of epidemic prevention in the long term, we increase the vaccination rate as carry over to construct Model 2 by Dynamic DEA as following [20]. The progress of the epidemic had an impact on the input and output of t + n period, meaning the input and output of each period would affect the next period through the carryover. As the purpose of this study was to explore the long-term performance of epidemic prevention policies, the dynamic DEA model was used to analyze the intertemporal efficiency and measure the overall national epidemic prevention efficiency. The flowchart is shown in Fig. 3. In Model 2, the numbers of confirmed cases and death were considered in the model. In Model 3 (shown in Fig. 4), we eliminate the variable of confirmed case for constructing the model that may investigate the effect of vaccine to the death. The dynamic DEA model is:

3 $$\:{min}\frac{\sum\:_{t=1}^{T}{W}^{t}\left[\frac{1}{m}{\sum\:}_{i=1}^{m}\left(1-\frac{{s}_{io}^{-t}}{{X}_{io}^{t}}\right)\right]}{\sum\:_{t=1}^{T}{W}^{t}\left[\frac{1}{s}{\sum\:}_{r=1}^{s}\left(1+\frac{{s}_{ro}^{+t}}{{y}_{ro}^{t}}\right)\right]}$$

$$\:s.t.\:\sum\:_{j=1}^{n}{\lambda\:}_{j}^{t}{X}_{ij}^{t}+{s}_{i}^{-t}={X}_{io}^{t}\:\:\:\:\:i=1,\dots\:,m,\:t=1,\dots\:,T$$

$$\:\sum\:_{j=1}^{n}{\lambda\:}_{j}^{t}{y}_{rj}^{t}-{s}_{r}^{+t}={y}_{ro}^{t}\:\:\:\:\:r=1,\dots\:,s,\:t=1,\dots\:,T$$

$$\:\sum\:_{j=1}^{n}{\lambda\:}_{j}^{t}{z}_{kj}^{(t,t+1)}\ge\:\sum\:_{j=1}^{n}{\lambda\:}_{j}^{t+1}{z}_{kj}^{(t,t+1)}\:\:\:\:\:k=1,\dots\:,p,\:t=1,\dots\:,T$$

$$\:{\lambda\:}_{j}^{t},\:{s}_{i}^{-t},\:{s}_{r}^{+t}\ge\:0$$

where $\:{\text{z}}_{\text{k}\text{j}}^{(\text{t},\text{t}+1)}$ shows $\:{K}^{th}$ output of $\:{j}^{th}$ at the time t, and enter to the time t + 1. $\:\sum\:_{j=1}^{n}{\lambda\:}_{j}^{t}=1$

### Clustering analysis

Clustering is an unsupervised learning algorithm that can be used to evaluate the differences and easy to understand the similarity between data [21]. Euclidean distance is a metric to find the similarity by calculating the distance between data and make subgroups. The data doesn’t to be labeled in advance. The formula is shown below [22]:

$$\begin{aligned} \:Euclidean\:distance\left(x,y\right)=&\sqrt{\sum\:({y}_{i}-{x}_{i}{)}^{2}}\:\:\:\:\:,\\&\:i=\text{1,2},\dots\:,n\end{aligned}$$

Within-Cluster Sum of Square (WCSS) is typical method that can score the results by the summation of the distance between data and centroid in all clusters. The formula is shown as following:

$$\begin{aligned} \:WCSS=&\sum\:_{c_i\in\:C}\sum\:_{{\overrightarrow p}_j\in\:c_i}\\&{\parallel{\overrightarrow p}_j-{\overrightarrow o}_j\parallel}_2\:,\:{\overrightarrow o}_j\in\:c_i\end{aligned}$$

where $\:{\overrightarrow{o}}_{j}$ is a centroid of every cluster, $\:{\overrightarrow{p}}_{j}$ is each data point in each cluster, and $\:{c}_{i}$ represents a final result of cluster [23].

## Results

In this study, the descriptive statistics was shown in Table 1. Between December 2020 to December 2021, the mean of deaths and confirmed cases were 7,631 and 1,028,521, in the 17 selected countries, respectively. The vaccination rate is 74.06% and the maximum of vaccination rate is 84.84%; however, the minimum is 51.01% only. There are significant differences among these countries.

**Table 1:** Descriptive Statistics of Variables

Variable (unit: number)	Maximum	Minimum	Mean	S.D.
Input				
Number of doctors	1,194,267	92,173	349,938	302,109
Number of nurses	1,003,000	227,292	993,610	979,138
Population density	527.30	3.32	158.78	158.77
Output				
Epidemic recovery (%)	0.9998	0.9549	0.9950	0.0058
Undesirable output				
Number of deaths	43,017	33	7,631	11,746
Number of confirmed cases	5,449,603	6,311	1,028,521	1,356,780
Carryover				
Vaccination rate (%)	84.84	51.01	74.06	10.03

*SD* Standard deviation

**Fig. 5:**
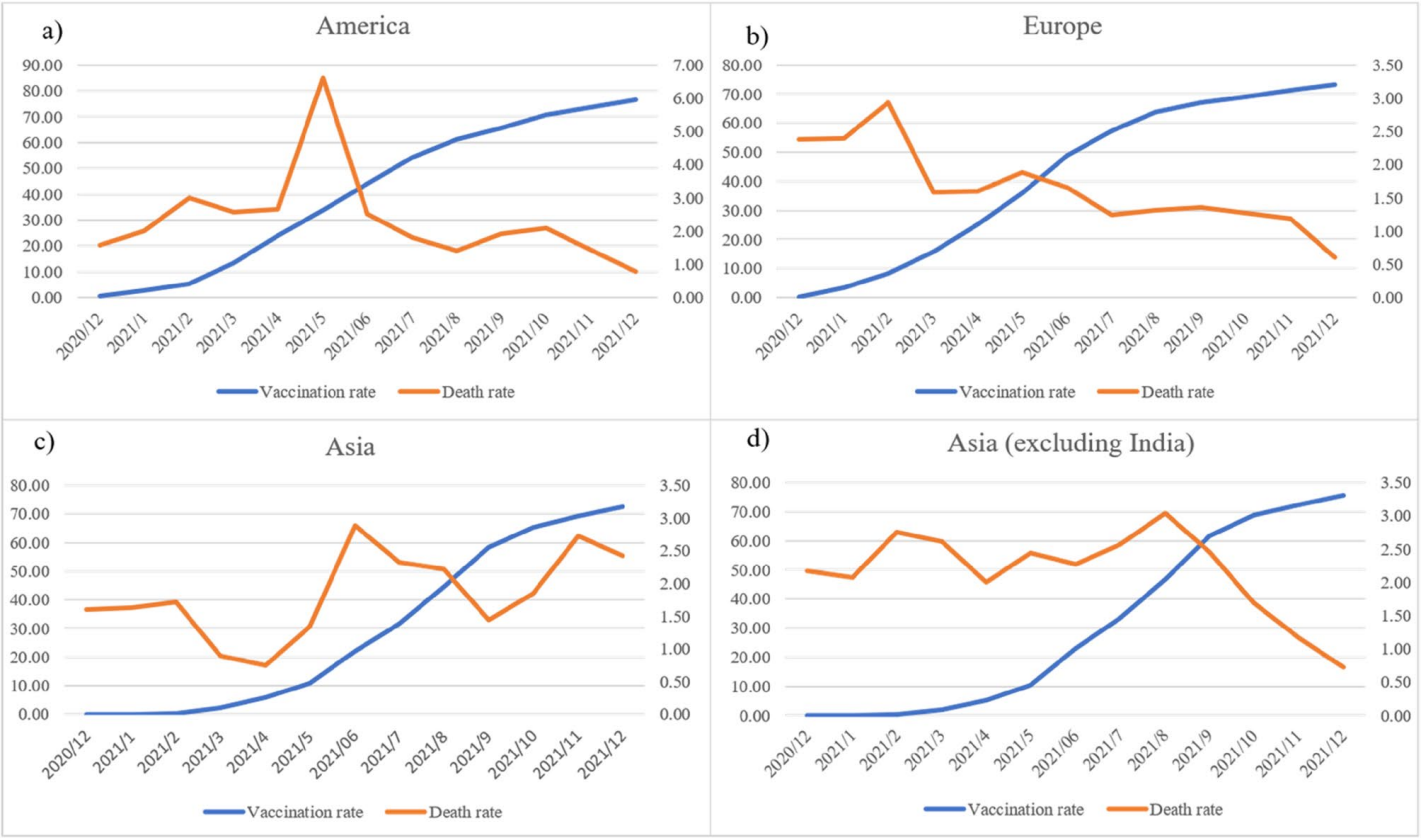
Vaccination Coverage and Mortality Rates from December 2020 to December 2021 **a** Americas **b** Europe **c** Asia (including India) **d** Asia (excluding India)

The trend of vaccination coverage and mortality rates in the Americas, Europe, and Asia are shown in Fig. 5. As can be seen from Fig. 5(a), the mortality rate in the Americas increased significantly in May 2021, which was influenced by the U.S.A. and Brazil, which were the second and third countries in the world with the largest increase in deaths caused by COVID-19. Although the U.S.A. had enough vaccines, the increase in deaths was due to the low coverage rate of vaccines in 2020; however, the number of deaths was brought under control when the vaccination rate exceeded 40%. According to Fig. 5(b), the mortality rate in Europe was well controlled due to an increased vaccination rate. Regarding Asia, as shown in Fig. 5(c), although the vaccination rate continued to rise, as in the Americas and Europe, India recorded the highest number of deaths in a single month in May 2021 (130,000), thus, the overall trend of mortality in Asia did not show the effect of the vaccine. In Fig. 5(d), we removed India from Asia and recalculated the vaccination and mortality rates, which showed that the vaccine was indeed effective in reducing the mortality rate.

**Fig. 6:**
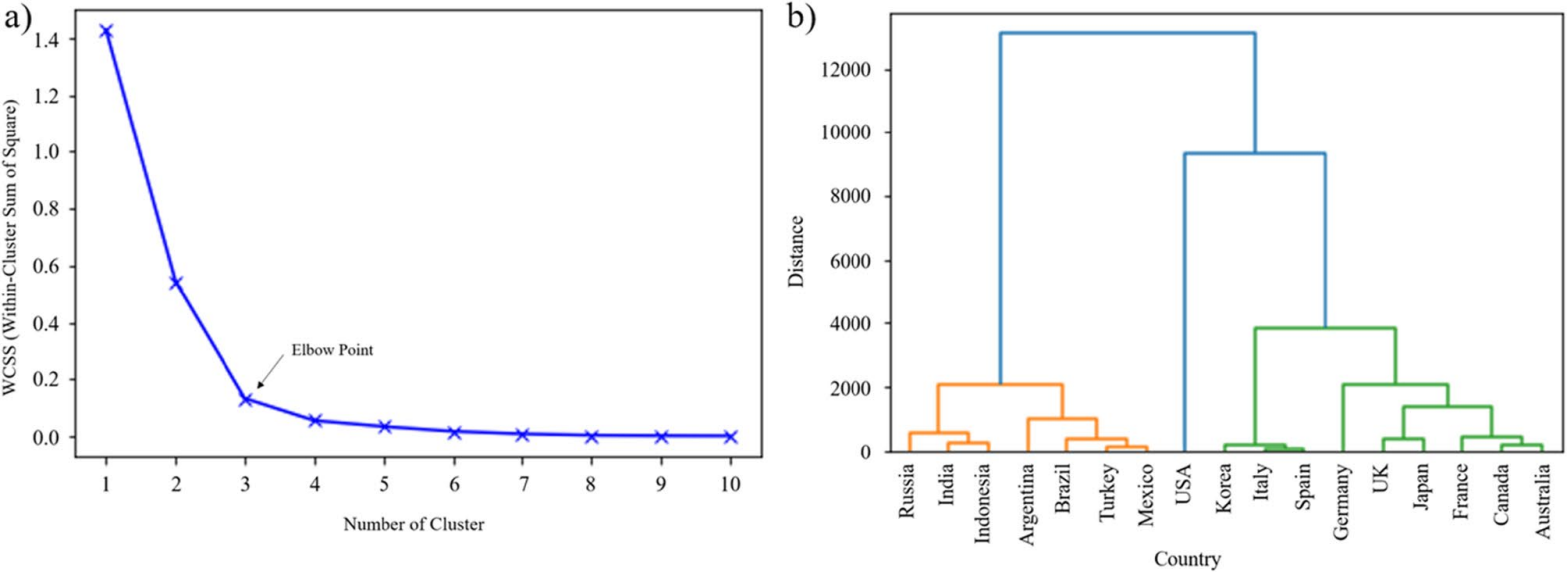
The clustering analysis of **a** elbow plot and **b** hierarchical cluster plot

We used the basic information to divide these countries into several groups via clustering analysis. Figure 6(a) showed the within-cluster sum of square (WCSS) and number of clusters. Three clusters were chosen as the best number of clusters based on the elbow point. Figure 6(b) illustrated the result of clustering analysis. According to the elbow point, we divided all countries into three clusters and showed below:

- Cluster 1: Russia, India, Indonesia, Argentina, Brazil, Turkey, and Mexico.
- Cluster 2: U.S.A.
- Cluster 3: Korea, Italy, Spain, Germany, UK, Japan, France, Canada, and Australia.

In the result, we observed that Cluster 1 and 3 had two countries with the best performance based on dynamic Data Envelopment Analysis model. In Cluster 1, Turkey and Argentina had the best performance. In Cluster 3, Canada and Australia ranked 1st. It indicated that the countries with similar characteristics can achieve the better performance in pandemic prevention by learning from the countries in the same cluster instead of from countries with different characteristics

Model 1 took the number of confirmed cases and the number of deaths as the undesired outputs, as shown in Table 2. It can be seen from Table 2 that the countries with poor epidemic prevention performance were mainly those in Europe and the U.S., as those countries did not closely observe social distancing, wearing masks, or other methods to prevent transmission. The main route of COVID-19 transmission was contact, which also led to the rapid spread of the COVID-19 in these countries.

**Table 2:** The effect of epidemic prevention

	**Model 1**	**Ranking**	**Model 2**	**Ranking**	**Model 3**	**Ranking**
Cluster 1						
Russia	0.2285	13	0.1254	10	0.1267	10
India	0.0602	17	0.0105	17	0.0101	17
Indonesia	0.4509	7	0.0781	14	0.0139	16
Argentina	1	1	1	1	1	1
Brazil	0.2186	14	0.0931	12	0.0729	13
Turkey	0.9613	4	1	1	1	1
Mexico	0.3470	9	0.0918	13	0.0726	14
Cluster 2						
U.S.A.	0.0603	16	0.0751	15	0.0835	12
Cluster 3						
Korea	0.4946	6	0.7794	5	0.4855	7
Italy	0.3502	8	0.3029	9	0.2838	9
Spain	0.6448	5	0.5970	6	0.5926	5
Germany	0.1532	15	0.1235	11	0.1006	11
UK	0.2623	11	0.4900	7	0.5298	6
Japan	0.3313	10	0.0463	16	0.0256	15
France	0.2292	12	0.3588	8	0.3770	8
Canada	1	1	1	1	1	1
Australia	1	1	1	1	1	1

With the advent of the vaccines, epidemic prevention focused on the administration of vaccines. Therefore, Model 2 regarded vaccines as the carry-over and used the dynamic Data Envelopment Analysis model to explore the effectiveness of vaccines in epidemic prevention over time. As the vaccination coverage rate increased, the average overall efficiency value of each period also gradually increased, indicating that the number of COVID-19 diagnoses and deaths were controlled by the vaccination rate, meaning vaccines could effectively reduce the negative outcome of the epidemic.

Model 3 focused on mortality as the key to epidemic prevention performance, thus, the number of confirmed cases was removed to evaluate the performance of epidemic prevention policies among countries, and the results showed that countries with higher vaccine coverage rates improved their epidemic prevention performance.

## Discussion

We selected 17 countries as the subjects in this study and Data Envelopment Analysis model was used to measure the efficiency performance of epidemic prevention policies. As the main modes of COVID-19 transmission were droplet transmission and direct contact, areas with high population density saw increased spreading of the disease. Medical personnel are an important medical resource during an epidemic, where the inputs are population density, the number of doctors, and the number of nurses, while the outputs are the number of confirmed cases, the number of deaths, and healthy life expectancy. This study constructed three models to explore the effects of epidemic prevention policies. Model 1 used the undesired output model, and took land area, population density, number of doctors, and number of nurses as inputs, the number of confirmed cases and deaths as undesired outputs, and healthy life expectancy as the normal output. Model 2 considered the effectiveness of vaccines against disease. The vaccination rates of various countries were included in the model, and the dynamic Data Envelopment Analysis model was used to explore the effect of time change on epidemic prevention through vaccines. Many documents have shown that vaccines can effectively reduce mortality[24–26]; therefore, Model 3 focused on the impact of vaccines on mortality, and excluded the number of confirmed cases. This study used data from December 2020 to December 2021 for analysis, meaning the period of one year after the global outbreak of COVID-19. When the COVID-19 vaccines were available to eligible people through EUA, although biotech companies planned to speed up vaccine production, production was still limited in its ability to meet the large global demand, thus, vaccines could not be administered on a large scale in most countries.

Compared to other countries, Model 1 showed that Europe and the U.S. have poor epidemic prevention performance. Su et al. [2] pointed out that the prevention efficiency rates of U.S., Brazil, and Russia were relatively low. Although entry bans on immigration and passengers from high-risk countries were announced at the beginning of the outbreak, there were loopholes in such policies. False negative results often occurred during the quarantine phase, which led to infected people returning home with the virus, tripling the actual number of infected people, or the importance of wearing masks was ignored. A survey in New York showed that if social distancing was mandatory when 100 people were infected, the maximum number of cases would have been 1,000, and if social distancing was mandatory when 200 people were infected, the number of cases would have been about 1,580 [27]. It was pointed out that the wearing of masks was inversely associated with COVID-19 infection rates, and that the number of new cases per week could be reduced by 25% if mask-wearing measures were implemented [28]. Therefore, countries accelerated the production of this health protective device, which were provided to front-line medical workers and distributed among international rescue networks. Some countries, such as Canada and Australia, implemented screening at the border, which achieved good results. In other words, in the early stage of the epidemic, isolating the source of the virus and implementing a screening policy achieved better epidemic prevention results, while the Americas and Europe were hardest hit by COVID-19. In order to solve the problem of the continuous growth of cases, governments must put forward specific intervention measures. After a period of intervention efforts and effective policies, as the people highly complied with such policies, some countries successfully and significantly improved their epidemic situation, while other countries were still at the peak of their epidemic situation or even faced continuous deterioration. This indicates that different prevention levels and stages of the epidemic were affected by different COVID-19 policies. The previous study [2] pointed out that the efficiency rates of the USA, Brazil, and Russia were relatively low. Although the entry bans on passengers, as announced at the beginning of the outbreak, was effective for preventing immigration cases from high-risk countries, there were loopholes in the policy. During the quarantine phase, false negatives often resulted in infected people returning home with the virus, which tripled the actual number of infected people, or they ignored the importance of wearing masks.

After the COVID-19 vaccine was introduced in the UK in December 2020, the desire to vaccinate increased around the world. The UK and Canada had the highest vaccination rates, and nearly 60% of the population in these two countries were vaccinated. Canada purchased large quantities of COVID-19 vaccines to ensure that all citizens could be fully vaccinated. The rankings by vaccination rate are USA, Germany, Italy, Spain, and France. At the end of 2020, the USA ranked second in the world, second only to the UK, but was overtaken by Canada in May 2021. People had doubts about vaccines and the government, thus, they held rigorous attitudes toward vaccine safety, had high autonomy, and believed that the virus was not aggressive. For these reasons, some people refused to be vaccinated, which negatively affected vaccine coverage, and many people ignored the risk of infection in their immediate surroundings. However, when the effects of vaccines were included in the model, countries with higher vaccination rates showed improvements in their epidemic prevention performance.

South Korea was once the hardest hit region in Asia. Specifically, more than 10,000 new cases were confirmed every day, and when the numbers of severe diseases and deaths skyrocketed, the South Korean government announced that the epidemic had entered its peak. In order to prevent further spread of the epidemic, intensive vaccination arrangements were made to increase the coverage rate to help South Korea successfully fight COVID-19. At the beginning of the outbreak, Australia announced the implementation of strict border control measures and actively planned vaccination programs. By the end of 2021, the vaccine coverage rate reached 80%, and the confirmed numbers of cases and deaths gradually slowed in Australia, and this result was attributed to the coverage rate of the vaccine. When faced with another attack of a variety of mutated strains, in order to strike a balance between economic openness and epidemic prevention policies, Australia no longer chose to lock down cities, but called on the public to take personal prevention measures and encouraged vaccination supplements. Indonesia was ravaged by the Delta mutant and the movement of people during Eid al-Fitr in May. In the face of its situation, Japan implemented entry control for some countries, but did not choose mandatory measures at home. Even though Japan's elderly population accounts for 30%, which makes it one of the top countries regarding its aging population, its death rate still did not peak. This was mainly because people highly complied with and were willing to observe the epidemic prevention policies, including wearing masks, reducing gatherings, avoiding close contact, and giving priority to vaccinating medical workers and the elderly. Therefore, Japan’s level of control in the early stage of the epidemic was relatively successful. With the opening of the 2020 Summer Olympics, sports athletes from various countries successively visited Japan; however, the rate of distribution for vaccination was delayed due to the shortage of imported vaccines. By mid-2021, only 30% of Japan's population had been vaccinated, while the vaccination rates in Canada, UK, USA, and Italy were 55% to 70% (shown in Table S1). Due to the uneven vaccination distribution, the news that the Olympic Committee workers, athletes, and media personnel were diagnosed was constantly reported, and some athletes announced their withdrawal from the competition. Even though the athletes had been administered with two doses of vaccine, most of the confirmed cases were breakthrough infections. The organizers did not cancel the Tokyo Olympics, which caused considerable concern, and with more than 1,000 people diagnosed every day [29], Japan finally decided to stop allowing spectators to watch the Olympic events, but the epidemic did not slow down, and the confirmed cases did not decline as expected [30]. The number of new infections overwhelmed Japan's medical capacity, and the public was dissatisfied with the government's anti-epidemic performance and vaccine progress, which led to severe criticism. This result shows that both vaccines and strict preventive measures were needed to stop the spread of COVID-19, and it was not optimistic to rely on vaccines alone. However, after the effectiveness of vaccines was included in the model, countries with more active vaccine coverage made progress in their epidemic prevention performance.

The selected 17 countries are from G20 countries, all of them have huge effect globally, including land, population and global GDP. However, these countries have different characteristics, e.g. Japan has the highest proportion of elderly population, America has more immigrants and world's top 50 pharmaceutical companies [35]. In the clustering analysis, the 17 countries are separated into three groups. However, only two cluster could be considered in this study after excluding America. In each group, there are two countries with the highest performance in efficiency. Each country can enhance their performance by learning from the countries. in the same group This is evidence that the process of quality improvement in healthcare highly reply on the country which patients and medical professionals in their local scenario [36].

By the end of 2020, biotechnology companies around the world had developed more than 150 COVID-19 vaccines to enable people to get vaccinated faster; however, some people had doubts about the safety and effectiveness of vaccines, which led to increased health risks for individuals and had adverse effects on the spread of COVID-19. In a severe epidemic situation, only a vaccine coverage rate of 60% can avoid community infection and play a protective role [37]. As Europe and the USA are the main regions for vaccine research and development, the vaccination rates in these countries were significantly higher than those in other regions. Therefore, the results clearly show that regions with high vaccination rates had improved epidemic prevention performance.

Since this study primarily focuses on the efficiency ranking among countries rather than determining the projection values, and considering the long-term analysis, we employ the dynamic DEA. However, in the dynamic DEA, the data presented in the projection cannot be ensured to be integers, which is a limitation of this study.

## Conclusions

The first outbreak of the COVID-19 in late 2019 swept across the world in a short period of time, and most countries quickly imposed strict controls on inbound passengers at airports and implemented epidemic prevention policies on a large scale. When faced with a sudden infectious disease, it is usually impossible to provide timely drug treatments or preventive vaccines, thus, only intervention policies can control the development of the epidemic in the early stage and prevent its spread in the short-term, and reduce inbound cases through border controls, while maintaining social distancing and home isolation, and wearing masks can reduce infections caused by contact. As the world learned more about the virus and the developed vaccines, we entered the post-epidemic era, when vaccine administration became the main epidemic prevention strategy. However, it does not mean that increased vaccination rates can completely replace preventive measures, and different new viruses will continue to emerge in the future. How to reduce the adverse impact on health and reduce the burden on the health care system will also become the goals of research. Therefore, in the post-epidemic era, governments should pursue how to reduce the mortality rate and maintain the operations of the medical system to ensure the health of the people, thus, continuing to administer vaccination boosters has become an important epidemic prevention policy, as regular vaccinations can reduce the probability of diagnosis, and effectively reduce the mortality rate.

## Supplementary Information

Supplementary Material 1

## Data Availability

The datasets generated and/or analyzed during the current study are available in the WHO repository, https://covid19.who.int/data (accessed on 10 March 2022).
