# Supplementary material for: Constructing protective mechanism by analysis of vaccine impact based on hybrid model of dynamic DEA and cluster tendency
Source: BMC Public Health. 2025 Oct 8;25:3392. doi: 10.1186/s12889-025-24277-9 (PMC12505740; doi:10.1186/s12889-025-24277-9)
Supplement: Supplementary file 1 — Supplementary Material 1 [file 12889_2025_24277_MOESM1_ESM.docx]

Supplementary Material

Table S1 The vaccine rate in 2021

|  | January | February | March | April | May | June | July | August | September | October | November | December |
| --- | --- | --- | --- | --- | --- | --- | --- | --- | --- | --- | --- | --- |
| USA | 9.01 | 16.94 | 32.78 | 46.12 | 52.30 | 55.28 | 58.21 | 62.31 | 64.88 | 66.88 | 70.42 | 73.52 |
| India | 0.27 | 0.84 | 3.94 | 8.85 | 11.80 | 19.15 | 25.39 | 35.07 | 45.79 | 51.76 | 55.68 | 59.67 |
| Brazil | 0.97 | 3.04 | 8.24 | 14.72 | 21.52 | 34.88 | 48.80 | 63.22 | 70.33 | 74.12 | 76.13 | 77.19 |
| Japan | 0.00 | 0.02 | 0.71 | 2.6 | 10.29 | 28.22 | 47.00 | 60.66 | 73.76 | 80.36 | 81.33 | 81.66 |
| Korea | 0.00 | 0.08 | 1.71 | 6.36 | 11.06 | 29.36 | 37.36 | 56.33 | 75.67 | 79.07 | 81.75 | 84.84 |
| Australia | 0.00 | 0.13 | 2.57 | 7.55 | 14.73 | 23.34 | 32.12 | 46.96 | 63.60 | 72.78 | 76.50 | 78.10 |
| Canada | 2.24 | 3.60 | 13.18 | 32.28 | 57.00 | 67.26 | 70.48 | 72.84 | 75.76 | 77.63 | 79.18 | 82.43 |
| Germany | 2.33 | 4.98 | 11.96 | 28.09 | 43.86 | 56.50 | 62.81 | 66.31 | 69.03 | 70.39 | 72.68 | 75.27 |
| Italy | 2.37 | 5.09 | 12.29 | 24.3 | 40.39 | 58.40 | 65.42 | 72.49 | 76.83 | 79.16 | 80.18 | 82.09 |
| Russia | 0.69 | 0.00 | 4.84 | 8.50 | 11.45 | 15.68 | 25.31 | 30.25 | 33.22 | 38.33 | 46.12 | 51.01 |
| Mexico | 0.50 | 1.48 | 5.42 | 9.79 | 17.10 | 24.71 | 36.76 | 45.33 | 50.13 | 58.51 | 60.27 | 64.25 |
| Indonesia | 0.18 | 0.61 | 2.94 | 4.50 | 5.96 | 10.63 | 17.14 | 22.91 | 33.06 | 43.43 | 50.59 | 58.47 |
| Argentina | 0.63 | 2.12 | 7.84 | 16.09 | 22.02 | 38.55 | 56.64 | 62.87 | 66.22 | 77.10 | 82.16 | 85.30 |
| Turkey | 0.00 | 8.05 | 10.54 | 16.07 | 19.49 | 41.12 | 48.06 | 56.56 | 63.09 | 64.91 | 65.93 | 66.69 |
| Spain | 2.63 | 5.40 | 11.17 | 24.73 | 38.22 | 53.89 | 66.97 | 76.82 | 79.28 | 79.98 | 80.65 | 83.40 |
| UK | 13.77 | 30.03 | 46.14 | 50.9 | 58.48 | 66.45 | 69.35 | 71.23 | 72.38 | 74.05 | 75.54 | 76.71 |
| France | 2.48 | 4.65 | 13.09 | 23.69 | 38.96 | 51.03 | 63.55 | 72.45 | 75.13 | 76.45 | 77.40 | 78.50 |

Source: Compiled by the authors based on WHO repository. https://covid19.who.int/data (accessed on 10 March 2022)
